# The impact of angiotensin-receptor neprilysin inhibitors on cardiovascular events and solute transport function in peritoneal dialysis patients: a multicenter retrospective controlled study

**DOI:** 10.1080/0886022X.2024.2431637

**Published:** 2024-11-28

**Authors:** Yishu Wang, Canxin Zhou, Xiaoyan Ma, Yingfeng Shi, Xiujuan Zang, Shoujun Bai, Yan Hu, Zexin Lv, Haijuan Hong, Yakun Wang, Danying Yan, Xinyu Yang, Chao Yu, Daofang Jiang, Shougang Zhuang, Yi Wang, Na Liu

**Affiliations:** a Department of Nephrology, Shanghai East Hospital, Tongji University School of Medicine, Shanghai, China; b Department of Nephrology, Shanghai Songjiang District Central Hospital, Shanghai, China; c Department of Nephrology, Qingpu Branch of Zhongshan Hospital Affiliated to Fudan University, Shanghai, China; d Department of Medicine, Rhode Island Hospital and Alpert Medical School, Brown University, Providence, RI, USA

**Keywords:** Peritoneal dialysis, angiotensin receptor-neprilysin inhibitor, cardiovascular events, peritoneal solute transport, neoangiogenesis

## Abstract

**Background:**

Whether angiotensin receptor-neprilysin inhibitor (ARNI) can reduce the incidence of cardiovascular events and improve peritoneal function in peritoneal dialysis (PD) patients remains unclear. Thus, this study aims to clarify the role of ARNI in PD patients.

**Methods:**

This was a multicenter retrospective study. A total of 102 patients were enrolled for analysis. Patients who continuously used ARNI for 12 months were assigned to the ARNI group (*n* = 55), while those who never used ARNI to the control group (*n* = 47). Clinical indicators and cardiovascular risk factors were analyzed, along with *in vitro* experiments on neoangiogenesis to investigate the underlying molecular mechanisms of peritoneal protection by ARNI.

**Results:**

Systolic blood pressure (*p* = 0.001), diastolic blood pressure (*p* = 0.001), and left ventricular ejection fraction (*p* = 0.008) were statistically improved after 12 months of ARNI therapy, whereas these metrics did not change in control patients. The risk factors for the occurrence of cardiac events in PD patients included the use of ARNI [hazard ratio (HR) 0.053; 95% confidence interval (CI), 0.006–0.492] and NT-proBNP level (HR 2.317; 95% CI, 1.179–4.554). Additionally, there was a decrease in 4-hour ratio of creatinine concentration in dialysate to plasma (4h Scr D/P) in the ARNI group (*p* = 0.020). The *in vitro* experiments showed that LCZ696, a combination of sacubitril and valsartan, inhibited neoangiogenesis *via* the VEGFR2/ERK1/2 and Notch1 pathways.

**Conclusions:**

ARNI may play a protective role in reducing the incidence of cardiovascular events and decreasing solute transport in PD patients.

## Introduction

Chronic kidney disease (CKD) is a common disease leading to a high prevalence of cardiovascular diseases and mortality, putting a heavy burden on society [1]. Peritoneal dialysis (PD) has emerged as a widely accepted form of renal replacement therapy since 1976. Currently, over 272,000 patients worldwide receive PD, accounting for more than 11% of all dialysis patients. Notably, the survival rate of PD is now comparable to that of hemodialysis [2].

Cardiovascular events and ultrafiltration failure are primary causes of death among patients undergoing maintenance PD [3]. Angiotensin receptor-neprilysin inhibitor (ARNI), previously known as LCZ696, is a novel combination drug with angiotensin receptor blocker and neprilysin inhibitor. Angiotensin receptor blockade can reverse cardiac remodeling by inhibiting members of the guanine nucleotide-binding protein family, while neprilysin inhibitor attenuates cardiomyocyte cell death, hypertrophy, and impaired myocyte contractility by inhibiting phosphatase and tensin homolog (PTEN) [4]. Previous studies have shown that ARNI has a good effect in lowering blood pressure, reducing proteinuria, and reducing the risk of death in heart failure patients [5–7]. However, patients receiving dialysis therapy were not included in these studies. In recent years, several studies have explored the cardioprotective effects of ARNI in dialysis patients. Ding et al. demonstrated that ARNI could improve ejection fraction in PD patients [8]. Fu et al. found that ARNI treatment could reduce NT-proBNP levels and improve heart failure symptoms in PD patients [9]. However, more clinical evidence is still needed to confirm these findings.

The success of long-term PD relies on adequate clearance of solutes and fluids. Peritoneal structure and function deteriorate with increased duration of PD, leading to ultrafiltration failure (UFF). Long-term exposure to high dextrose concentration dialysate, numerous toxins accumulating and peritonitis all induce excessive angiogenesis and mesothelial cell loss and finally lead to technique failure [10]. Angiogenesis is one of the major mechanisms of UFF [11]. In recent years, extensive research has been conducted on the underlying mechanisms regulating peritoneal angiogenesis. Our previous study indicates that vascular endothelial growth factor (VEGF) expression levels are significantly elevated in the dialysis effluent of PD patients with long-term PD [12]. Another study has confirmed that patients with elevated expression of VEGF-A often have a higher dialysate/plasma (D/P) ratio, and VEGF-A concentration expression levels are positively correlated with the number of blood vessels and peritoneal thickness [13]. A prospective, randomized, double-blind trial showed that high-dose trans-resveratrol, a natural polyphenolic compound known for its antioxidant, anti-inflammatory, and cardioprotective properties, could improve peritoneal ultrafiltration by reducing angiogenesis through the lowering of VEGF [14]. Other studies related to PD have also confirmed that blocking signaling pathways such as vascular endothelial growth factor receptor 2 (VEGFR2)/extracellular signal-regulated protein kinase 1/2 (ERK1/2) and Notch, which influence angiogenesis, can downregulate angiogenesis and improve peritoneal ultrafiltration function [15–19]. However, it remains unknown whether ARNI can improve neovascularization and peritoneal function in PD patients through these pathways until now.

In this context, we conducted a retrospective study of PD patients treated with ARNI for 12 months and explored the potential effects of ARNI on the peritoneum through *in vitro* experiments. This study aims to evaluate the impact of ARNI on cardiovascular prognosis and peritoneal function, providing a theoretical basis for developing better treatment strategies for PD patients and offering new clinical treatment options.

## Materials and methods

### Study design and participants

**Figure 1.**
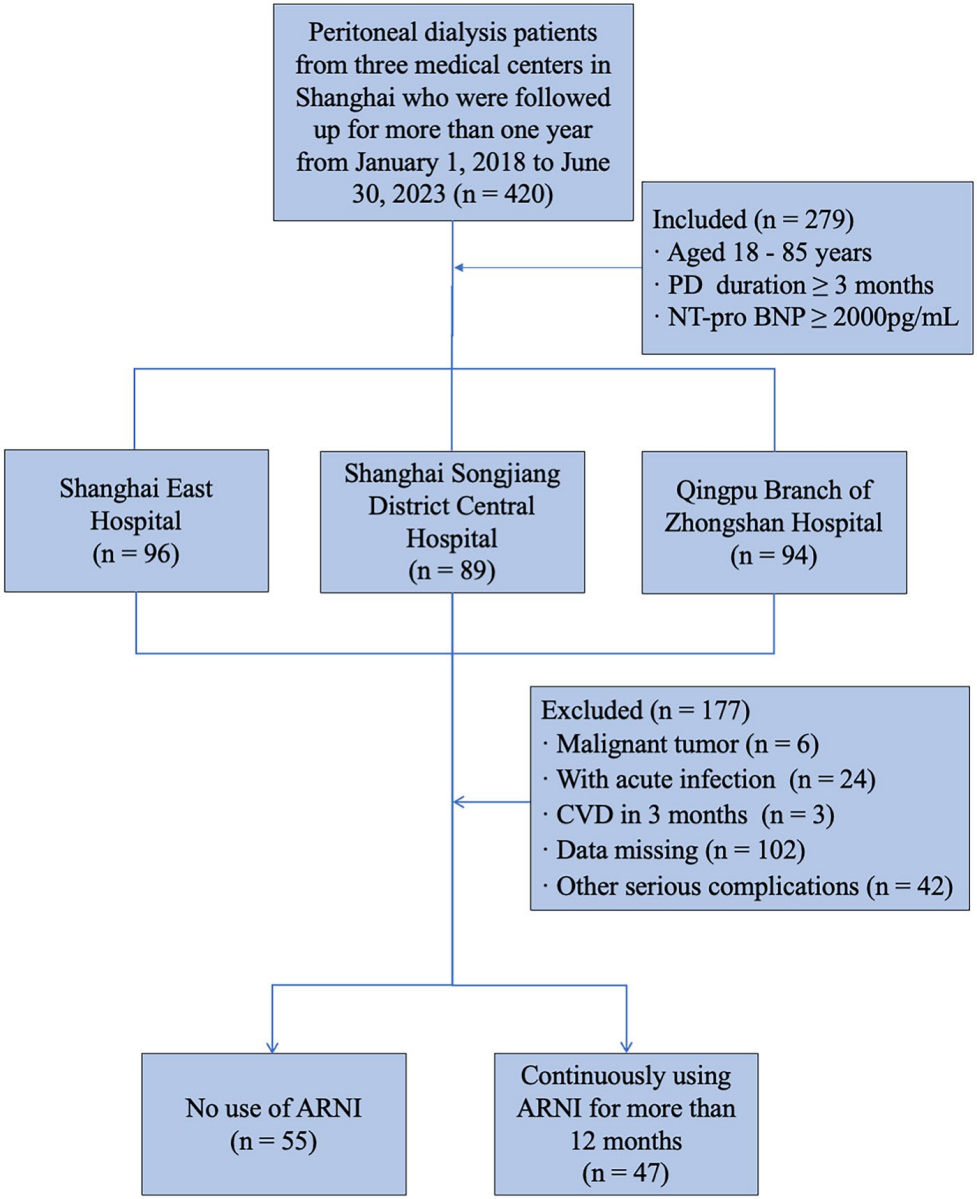
Flowchart of the ARNI study. A total of 279 peritoneal dialysis patients from 3 centers in Shanghai were included in the study, and 177 patients were excluded according to the exclusive criteria. 102 patients were finally enrolled, consisted of 55 patients who had not used ARNI and 47 patients treated with ARNI for more than 12 months. Abbreviations: PD, peritoneal dialysis; NT-proBNP, N-terminal pro-B-type natriuretic peptide; ARNI, angiotensin receptor-neprilysin inhibitor; CVD, cardiovascular disease.

This multicenter retrospective study recruited PD patients who were followed up for more than 1 year and had complete data from January 1, 2018 to June 30, 2023 in Shanghai East Hospital, Shanghai Songjiang District Central Hospital, and Qingpu Branch of Zhongshan Hospital. Patients who met the following criteria were included in the study: patients aged between 18 to 85 years old; patients on PD treatment for more than 3 months. Due to the limitations of the testing conditions, we could only select N-terminal pro-B-type natriuretic peptide (NT-proBNP) as an observation indicator instead of B-type natriuretic peptide (BNP). NT-proBNP was not affected by neprilysin inhibitors, so its concentration decreased with effective treatment [20]. Additionally, NT-proBNP was more stable and had significant diagnostic value for heart failure. According to previous systematic review and meta-analysis, patients who had NT-proBNP over 2,000 pg/mL often had higher risk of cardiovascular events, and as NT-proBNP levels increased, the risk of cardiovascular events also increased [21]. Based on the reference, we chose NT-proBNP > 2,000 pg/mL as inclusion criteria. The exclusion criteria included patients with malignant tumors, acute infections, cardiovascular events within the past 3 months, missing data, and those with other serious complications including abnormal liver function, such as alanine aminotransferase (ALT) and/or aspartate aminotransferase (AST) levels 2–3 times the upper limit of normal, total bilirubin more than 2 times the upper limit of normal and history of major surgery or trauma within the last 3 months. To maintain consistency in the study protocol and minimize potential confounding variables associated with the different dialysis modalities, we also excluded patients receiving automated peritoneal dialysis (APD). All patients enrolled in this study received continuous ambulatory peritoneal dialysis (CAPD) using 1.5% or 2.5% glucose-containing dialysis fluid (Baxter Healthcare, Guangzhou, China), which was exchanged 3 to 5 times daily and underwent a peritoneal equilibration test (PET) every 6 months. In this study, ARNI therapy was primarily prescribed by cardiologists based on the patient’s blood pressure, cardiac function, overall health condition, and comorbidities. Patients were divided into 2 groups: the ARNI group in which patients continued to use ARNI for at least 12 months; and the control group in which patients didn’t use ARNI at all (Figure 1). The study protocol was approved by the Human Research Ethics Committee of Shanghai East Hospital Affiliated with Tongji University School of Medicine (ChiCTR2200064883).

### Data collection

The baseline demographic data were collected from the electronic medical record system. Clinical biochemical parameters included hemoglobin (Hb), serum albumin (Alb), blood glucose (BG), blood urea nitrogen (BUN), serum creatinine (Scr), uric acid, estimate glomerular filtration rate (eGFR), ALT, AST, glycated hemoglobin A1c (HbA1c), intact parathyroid hormone (iPTH), fasting glucose, high-density lipoprotein (HDL), low-density lipoprotein (LDL), total cholesterol (TC), triglyceride and NT-proBNP at baseline and 12 months.

Twenty-four-hour urine and PD effluent samples were collected at baseline and 12 months for measurement of weekly urea clearance (Kt/V). Data from standard 4-h peritoneal equilibration test at baseline and 12 months were collected. PD effluent samples were collected at 0, 2, and 4 h, venous blood samples were collected at 2h for evaluation of peritoneal transport characteristics. According to the 4-h ratio of creatinine concentration in dialysate to plasma (4h Scr D/P), peritoneal transport status was categorized as low (L), low average (LA), high average (HA) and high (H) (*L* < 0.50, LA 0.5 − 0.64, HA 0.65 − 0.80, *H* ≥ 0.81). Cardiac structure and function were assessed by two-dimensional echocardiography.

### Outcomes

Primary outcomes were occurrences of cardiovascular events after 12-month follow-up. Cardiovascular events were determined as congestive heart failure (CHF), ST-segment elevation myocardial infarction (STEMI) or non-STEMI. Secondary outcomes included changes in clinical indicators such as blood pressure, NT-proBNP, cardiac structure and function, peritoneal function and residual renal function after 12-month follow-up.

### Cell culture and treatments

The human umbilical vein endothelial cells (HUVECs) were obtained from the American Type Culture Collection (ATCC, Manassas, VA, USA). Vascular endothelial growth factor (VEGF) protein was purchased from R&D Systems (Minneapolis, MN, United States). HUVECs were grown in high glucose medium supplemented with 10% fetal bovine serum (FBS) and 1% penicillin/streptomycin in a 5%CO2 humidified incubator at 37 °C. HUVECs were starved for 24 h with high glucose medium containing 0.5% FBS and then pretreated with or without LCZ696 (ARNI, 25 μM) for 1 h and then coincubated with or without VEGF (25 ng/mL) for 24 h before cell harvesting. All of the *in vitro* experiments were repeated for no less than 3 times.

### Immunoblot analysis

Antibodies to p-VEGFR2 (#2478), VEGFR2 (#9698), p-ERK1/2 (#4370), ERK1/2 (#4695), Notch1 (#3608) were purchased from Cell Signaling Technology. Identical amount of total protein from the culture HUVECs were denatured and the proteins of each group were performed with immunoblotting by using the appropriate antibodies [22]. The expression level was determined by measurement of the corresponding band intensities using ImageJ software (National Institutes of Health, Bethesda, MD, USA).

### Tube formation assay

Matrigel was pipetted into a 24-well plate and solidified at 37 °C for 30 min. HUVECs were seeded into the Matrigel and cultured in high glucose medium with or without LCZ696 (25 μM) and coincubated with or without VEGF (25 ng/mL). After 12 h, tube formation was viewed and images were captured, the quantitatively analyzed for total segment length were used by ImageJ software.

### Wound healing assay

Firstly, HUVECs were seeded in 6-well plates until they reach 90% confluence. The cells were scratched using a 200 µl pipette tip and a wound was formed. The detached cells were washed with PBS for 3 times. The HUVECs were cultured in serum-free high glucose medium with or without VEGF (25 ng/mL) and LCZ696 (25 μM). Wounded cells were immediately photographed using an inverted light microscope (Zeiss Vert A1) at 0 h and then incubated for 36 h and imaged again. The width of the wound was measured by ImageJ software (National Institutes of Health, Bethesda, MD, USA). The migratory rate was calculated as (A − B)/*A* × 100%, where A and B reflect the width of the wound at 0 and 36 h, respectively.

### Statistical analysis

All statistics were analyzed using SPSS 26.0 and the statistical charts were described by GraphPad Prism 8.0. Continuous variables with normal distribution were presented as mean ± standard deviation (SD) while abnormal distribution was described as the median with the interquartile range (IQR). For normally distributed quantitative data, independent samples t-tests and paired samples t-test was employed to compare self-matching data. For non-parametric data, the Wilcoxon matched-pair signed-rank (two samples) test was applied. The chi-square test and Fisher’s exact test were used to identify any significant association between categorical variables.

Kaplan-Meier survival analysis was used to analyze patients’ prognoses. Univariate and multivariate Cox proportional hazards models were used to identify prognostic risk factors associated with cardiovascular events. Results were expressed as hazard ratios (HR) and their corresponding 95% confidence intervals (CI). *p* < 0.05 was considered statistically significant. In the univariate Cox regression analysis, all risk factors with a *p* value less than 0.2 were included in the multivariate Cox regression analysis.

*In vitro* experimental section, all the experiments were conducted at least 3 times. Data depicted in the graphs represent the means ± SEM for each group. One-way ANOVA was used for comparisons between groups. Tukey’s test was used for comparison of multiple groups’ means. Student’s t-test was used for differences between the two groups. All the results with *p* < 0.05 were considered statistically significant.

## Results

### Baseline characteristics

Of a total of 420 patients initially screened, 102 patients were eventually enrolled per the inclusion and exclusion criteria, with 47 in the ARNI group and 55 in the control group. Among the 102 patients, the mean age was 61.45 ± 14.81 years in the ARNI group and 56.81 ± 13.24 years in the control group. Of these patients, 63 (61.8%) were male, 46 (45.10%) had diabetes, 101 (99%) had hypertension and 26 (25.5%) had cardiovascular disease. The median duration of PD was 21.06 (IQR 8.00, 37.00) months in the ARNI group and 7.07 (IQR 5.00, 24.00) months in the control group. A total of 35 (63.6%) patients in the control group were treated with angiotensin-converting enzyme inhibitor or angiotensin II receptor blocker (ACEI/ARB). The demographic characteristics and the prevalence of diabetes, hypertension, and atherosclerotic vascular disease were similar in both groups. Both systolic blood pressure (*p* = 0.044) and diastolic blood pressure (*p* = 0.043) were significantly higher in the ARNI group than in the control group. In terms of clinical indicators, there were no statistically significant differences between the 2 groups except for the duration of PD (*p* = 0.043), SBP (*p* = 0.044), DBP (*p* = 0.043), Alb (*p* = 0.007) and left ventricular ejection fraction (LVEF) (*p* = 0.023). The baseline demographic and clinical characteristics are shown in Table 1. The effect of peritoneal dialysis regimen adjustments on residual renal function and volume in the ARNI subgroup during the one-year observation period are shown in Table 2.

**Table 1.** Baseline characteristics between the ARNI and the control groups.

Variables	Control (*n* = 55)	ARNI (*n* = 47)	*p* value*
Age, years	61.45 ± 14.81	56.81 ± 13.24	0.100
Sex, male, n (%)	33 (60.0)	30 (63.8)	0.692
Mean BMI, kg/m^2^	23.49 ± 3.33	23.71 ± 4.05	0.758
Duration of PD, months	21.06 (8.00, 37.00)	7.07 (5.00, 24.00)	**0.043**
SBP, mmHg	144.98 ± 30.16	156.36 ± 25.50	**0.044**
DBP, mmHg	80.91 ± 14.31	86.89 ± 15.20	**0.043**
Disease history, n (%)			
Diabetes	21 (38.2)	25 (53.2)	0.163
Hypertension	54 (98.2)	47 (100.0)	0.129
Cardiovascular disease	15 (27.3)	11 (23.4)	0.655
Medications			
Calcium channel blockers, n (%)	46 (83.6)	39 (83.0)	0.929
β-blockers, n (%)	22 (40.0)	21 (44.7)	0.633
α-blockers, n (%)	16 (29.1)	18 (38.3)	0.325
Diuretic, n (%)	14 (25.4)	12 (25.5)	0.993
ACEI/ARB, n (%)	35 (63.6)	0 (0.0)	–
Dialysis parameters			
Total weekly Kt/V	1.89 ± 0.36	1.82 ± 0.42	0.386
Weekly PD Kt/V	1.47 ± 0.36	1.52 ± 0.41	0.603
Weekly renal Kt/V	0.34 (0.00, 0.70)	0.20 (0.00, 0.56)	0.217
Ultrafiltration, mL	600.00 (400.00, 900.00)	600.00 (260.00, 1010.00)	0.810
Urinary output, mL	400.00 (0.00, 800.00)	500.00 (0.00, 900.00)	0.910
4h Scr D/P	0.67 ± 0.11	0.69 ± 0.13	0.385
The number of 2.5% dialysis solution exchanges	1.00 (0.00, 2.00)	1.00 (0.00, 2.00)	0.361
The number of 1.5% dialysis solution exchanges	2.00 (1.50, 2.50)	3.00 (2.00, 3.00)	0.079
Hb, g/L	99.23 ± 18.68	102.09 ± 22.77	0.490
Alb, g/L	33.73 ± 4.86	31.32 ± 3.72	**0.007**
Scr, μmol/L	856.73 ± 252.26	890.96 ± 302.87	0.537
SUA, μmol/L	386.96 ± 90.26	359.84 ± 101.91	0.164
BUN, mmol/L	19.42 ± 5.06	19.84 ± 5.94	0.705
eGFR, mL/min/1.73 m^2^	4.86 (4.02, 6.33)	4.52 (3.57, 6.14)	0.764
ALT, U/L	12.00 (9.00, 18.18)	13.00 (8.95, 18.00)	0.940
AST, U/L	17.00 (13.00, 22.00)	16.00 (11.40, 21.25)	0.419
Fasting glucose, mmol/L	5.25 (4.70, 6.85)	5.77 (5.00, 7.91)	0.093
HbA1c, %	6.35 ± 1.53	6.49 ± 1.34	0.647
Calcium, mmol/L	2.30 ± 0.49	2.19 ± 0.25	0.171
Phosphorus, mmol/L	1.58 ± 0.45	1.73 ± 0.71	0.213
iPTH, pmol/L	134.00 (81.22, 382.20)	156.55 (94.45 310.48)	0.951
HDL-cholesterol, mmol/L	1.02 ± 0.28	1.00 ± 0.34	0.753
LDL-cholesterol, mmol/L	2.43 ± 1.02	2.44 ± 1.01	0.951
TC, mmol/L	4.02 (2.78, 5.56)	3.97 (3.24, 4.99)	0.613
TG, mmol/L	1.74 (1.26, 3.03)	1.39 (1.04, 1.95)	0.060
NT-proBNP, pg/mL	6,080.00 (3677.00, 14055.50)	6,521.00 (3574.00, 20700.00)	0.445
Cardiac structure			
LVEF, %	62.11 ± 6.37	58.23 ± 8.67	**0.023**
IVS, mm	10.35 ± 1.84	10.55 ± 1.98	0.652
LVDs, mm	34.10 ± 5.08	34.18 ± 5.89	0.948
LVDd, mm	49.35 ± 9.54	50.32 ± 5.88	0.590
LAD, mm	40.26 ± 7.53	40.73 ± 5.96	0.764
AOD, mm	30.39 ± 3.86	30.82 ± 3.18	0.643

Quantitative data was expressed as median with interquartile range (IQR) or mean ± standard deviation (SD), categorical data was presented as frequencies and percentages [n (%)]. Bold indicates significance at *p* < 0.05.

Abbreviations: ARNI, angiotensin receptor-neprilysin inhibitor; BMI, body mass index; SBP, systolic blood pressure; DBP, diastolic blood pressure; PD, peritoneal dialysis; ARB, angiotensin II receptor blocker; ACEI, angiotensin-converting enzyme inhibitor; 4h Scr D/P, 4-h ratio of creatinine concentration in dialysate to plasma; Weekly Kt/V, weekly fractional clearance index for urea; Hb, Hemoglobin; Alb, albumin ; BUN, blood urea nitrogen; Scr, serum creatinine; SUA, serum uric acid; iPTH, intact parathyroid hormone; eGFR, estimate glomerular filtration rate; HDL, high-density lipoprotein; ALT, alanine transaminase; AST, aspartate aminotransferase; HbA1c, glycated hemoglobin A1c; LDL, low-density lipoprotein; TC, total cholesterol; TG, triglyceride; NT-proBNP, N-terminal pro-B-Type Natriuretic Peptide ; LVEF, left ventricular ejection fractions; IVS, interventricular septum; LVDs, left ventricular end-systolic dimension; LVDd, left ventricular end-diastolic dimension; LAD, left atrium diameter; AOD, aortic root diameter.

* The difference in the change between the ARNI and the control groups.

### Outcomes

**Figure 2.**
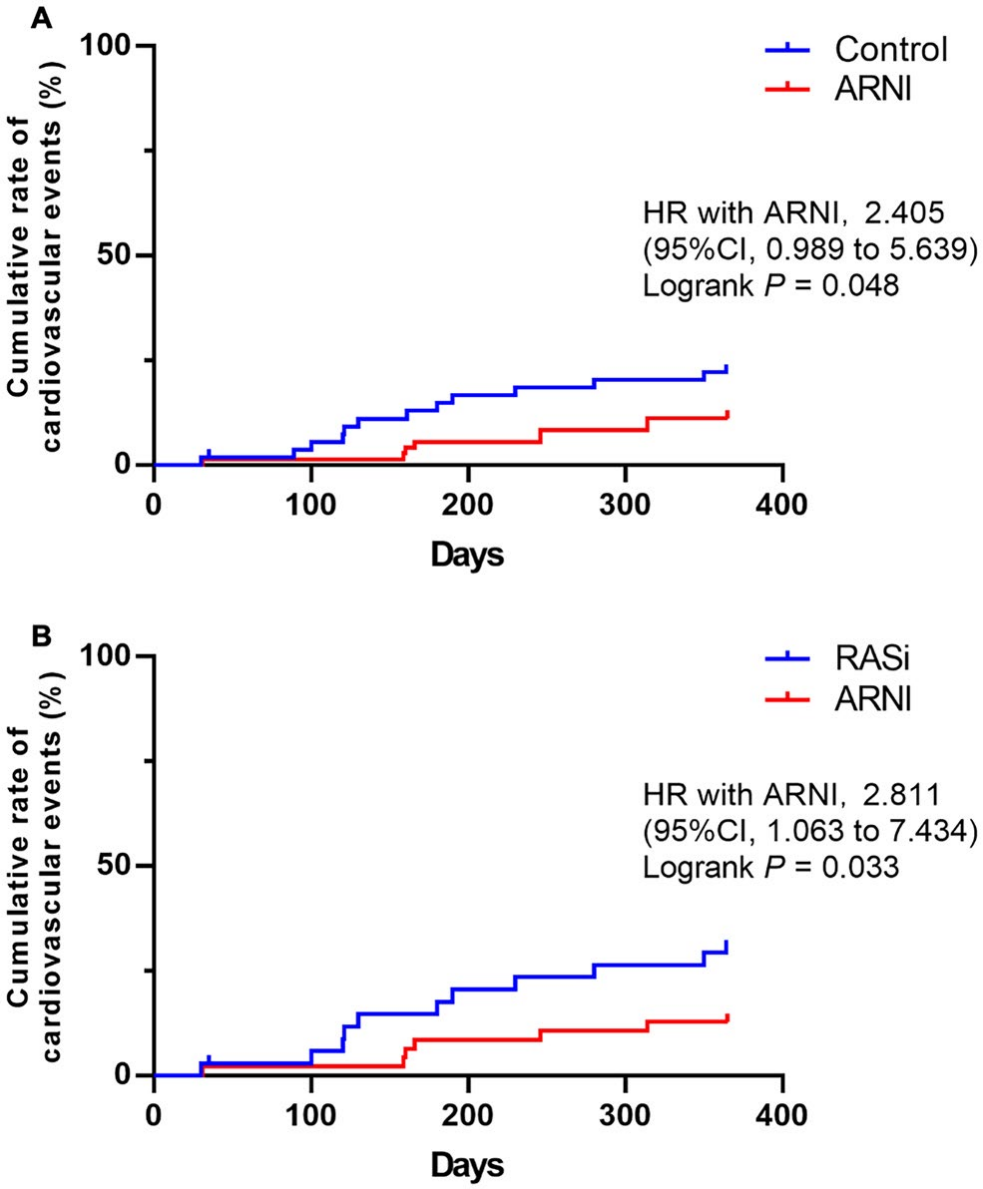
Kaplan-Meier curves for cardiovascular events in patients with or without ARNI. Kaplan-Meier curves were shown for the cumulative rate of cardiovascular events during the follow-up period in patients in both groups. (A) The cumulative rate of cardiovascular events during the follow-up period in patients in control group compared with the patients in ARNI group; (B) the cumulative incidence of cardiovascular events during the follow-up period in patients in the RASi-treated subgroup of the control group compared with the patients in ARNI group. Abbreviations: ARNI, angiotensin receptor-neprilysin inhibitor; RASi, renin-angiotensin system inhibitors; 95% CI, 95% confidence interval; HR, hazard ratio. *p* < 0.05 was considered to be statistically significant.

During the 12-month observation period, a total of 19 patients experienced cardiovascular events, with 13 in the control group and 6 in the ARNI group. There was a significant difference in the cumulative rate of cardiovascular events between the two groups (HR 2.405, 95%CI 0.989–5.639, *p* = 0.048) (Figure 2(A)). In the subgroup analysis, during the 12-month observation period, a total of 16 patients experienced cardiovascular events, with 11 in the control group treated with renin-angiotensin system inhibitors (RASi) and 6 in the ARNI group. The incidence of cardiovascular events was lower in the ARNI group compared to the RASi group (HR 2.811, 95%CI 1.063 − 7.434, *p* = 0.033) (Figure 2(B)).

In univariate Cox proportional hazards model analyses, age (HR 1.042, 95% CI 1.005 to 1.081, *p* = 0.025) and diabetes (HR 2.745, 95% CI 1.043 to 7.225, *p* = 0.041) were both risk factors for the occurrence of cardiovascular events. After ­multivariate adjustment, ARNI (HR 0.053, 95% CI 0.006 to 0.492, *p* = 0.010) and NT-proBNP (HR 2.317, 95% CI 1.179 to 4.554, *p* = 0.015) were significant independent factors for ­cardiovascular events (Table 3).

**Table 2.** The effect of peritoneal dialysis regimen adjustments on residual renal function and volume in the ARNI subgroup during the one-year observation period.

	Without adjustments		*p* value*	With adjustments		*p* value*
	Baseline	12-month		Baseline	12-month	
NT-proBNP	5,959.50 (2206.00, 9713.00)	3,227.00 (198.00, 6256.00)	**0.005**	11,720.50 (3451.00, 25197.00)	9,300.00 (555.00, 20253.00)	0.959
Weekly renal Kt/V	0.33 (0.06, 0.61)	0.19 (0.00, 0.52)	0.289	0.59 (0.35, 0.82)	0.22 (0.00, 0.72)	0.600

Quantitative data was expressed as median with interquartile range (IQR). Bold indicates significance at *p* < 0.05.

Abbreviations: ARNI, angiotensin receptor-neprilysin inhibitor; NT-proBNP, N-terminal pro-B-type natriuretic peptide; weekly renal Kt/V, weekly renal fractional clearance index for urea.

* The difference in volume and residual renal function between baseline and after 12-month of peritoneal dialysis regimen adjustment in ARNI subgroup.

### The effect of ARNI on volume and echocardiographic parameters

**Figure 3.**
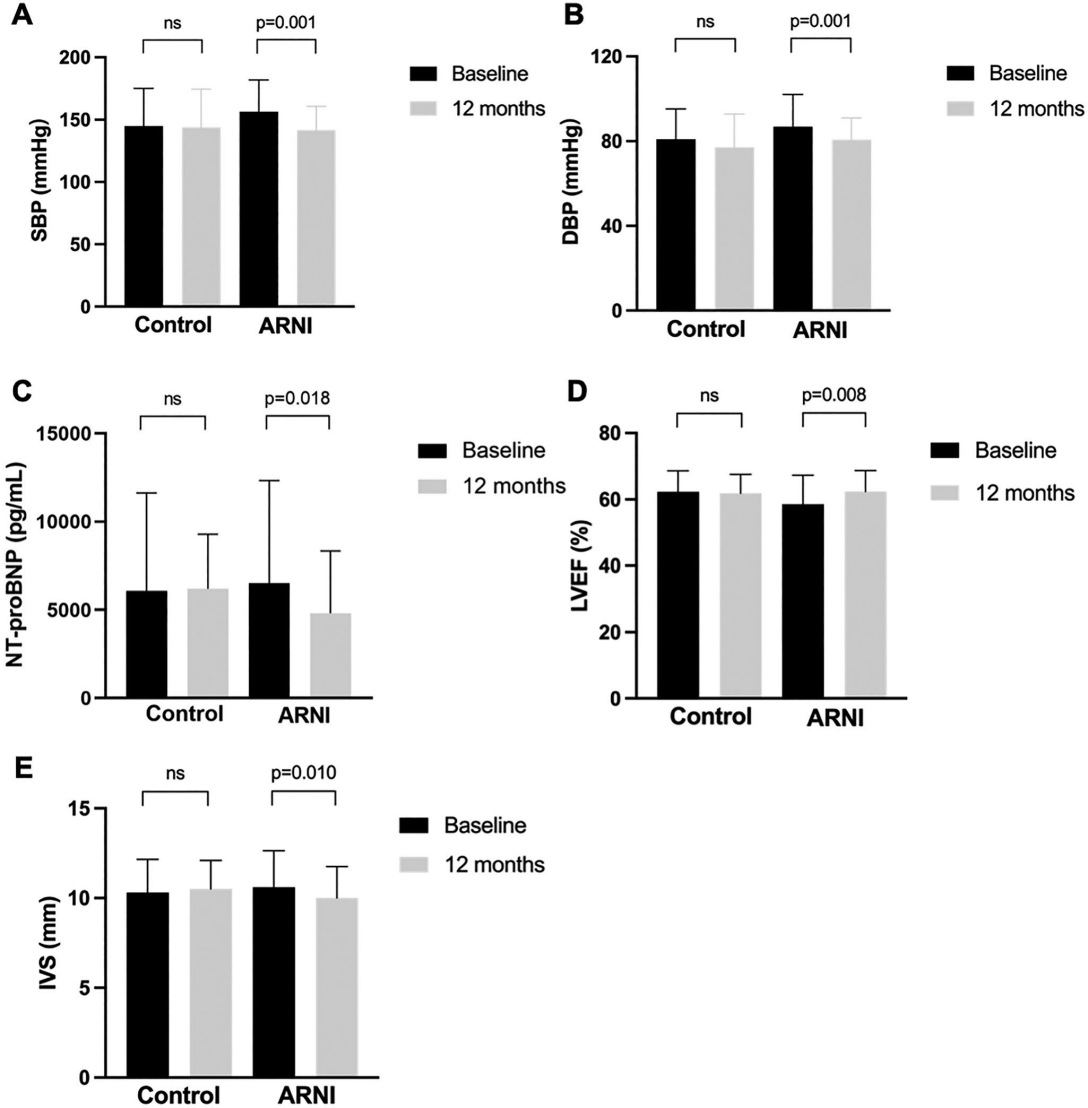
Changes in cardiac function and volume measures in the control group and the ARNI group at baseline and at 12 months. The histograms show the changes in cardiac function and volume measures at baseline and 12 months in both groups. (A) The change in SBP at baseline and 12 months in both groups. (B) The change in DBP at baseline and 12 months in both groups. (C) The change in NT-proBNP at baseline and 12 months in both groups. (D) The change in LEVF at baseline and 12 months in both groups. (E) The change in IVS at baseline and 12 months in both groups. Mean ± SD presented for variables according with normal distribution while median with IQR presented for variables with abnormal distribution. Abbreviations: DBP, diastolic blood pressure; SBP, systolic blood pressure; IVS, interventricular septum; LEVF, left ventricular ejection fraction; NT-proBNP, N-terminal pro-B-type natriuretic peptide; *p* < 0.05 was considered to be statistically significant.

After 12 months of ARNI treatment, the median SBP decreased from 156 mmHg to 142 mmHg (*p* = 0.001). The median DBP decreased from 87 mmHg to 81 mmHg (*p* = 0.001). The median NT-proBNP level decreased from 6521.00 pg/mL to 4817.00 pg/mL (*p* = 0.018). In the ARNI subgroup analysis, adjustments of the PD regimen did not result in significant changes in NT-proBNP levels (*p* = 0.959); but patients with no adjustments showed significant decrease in NT-proBNP level (*p* = 0.005) (Table 2). In terms of cardiac function, the mean LVEF increased from 58.56% to 62.34% (*p* = 0.008) and the interventricular septum (IVS) thickness decreased from 10.59 mm to 10.00 mm (*p* = 0.010). However, there were no statistically significant changes in the control group (Figure 3).

**Table 3.** Predictors of the cardiovascular events based on univariate and multivariate cox regression analysis.

Variables	Univariate			Multivariate		
	β	*p* value	HR (95%CI)	β	*p* value	HR (95%CI)
Age (per year)	0.041	**0.025**	1.042 (1.005–1.081)			
Sex						
Female	Ref	–	–			
Male	0.043	0.927	1.043 (0.411–2.653)			
Duration of PD, months	0.003	0.629	1.003 (0.990–1.017)			
Diabetes						
No	Ref	–	–			
Yes	1.010	**0.041**	2.745 (1.043–7.225)			
Cardiovascular disease						
No	Ref	–	–			
Yes	0.370	0.453	1.448 (0.550–3.809)			
ARNI						
No	Ref	–	–			
Yes	−0.698	0.157	0.498 (0.189–1.309)	2.942	**0.010**	0.053 (0.006–0.492)
Hemoglobin, g/L	−0.013	0.298	0.987 (0.964–1.011)			
iPTH, pmol/L	0.001	0.072	1.00 (1.000–1.003)			
Alb, g/L	0.012	0.784	1.012 (0.928–1.104)			
Scr, μmol/L	−0.001	0.194	0.999 (0.997–1.001)			
BUN, mmol/L	−0.042	0.262	0.959 (0.892–1.032)			
SUA, μmol/L	0.001	0.728	1.001 (0.996–1.006)			
TC, mmol/L	−0.011	0.758	0.989 (0.923–1.061)			
TG, mmol/L	−0.008	0.748	0.992 (0.945–1.041)			
LDL-cholesterol, mmol/L	−0.012	0.770	0.988 (0.911–1.072)			
HbA1c, %	0.214	0.111	1.239 (0.952–1.612)			
NT-pro BNP, pg/mL	0.341	0.107	1.407 (0.928–2.131)	0.840	**0.015**	2.317 (1.179–4.554)
Total weekly PD Kt/V	1.268	0.065	3.553 (0.922–3.685)			
Total weekly renal Kt/V	−0.086	0.731	0.917 (0.561–1.499)			
LVEF, mm	0.056	0.168	1.057 (0.977–1.145)			
IVS, mm	−0.232	0.142	0.793 (0.581–1.081)			

Bold indicates significance at *p* < 0.05.

Abbreviations: ARNI, angiotensin receptor-neprilysin inhibitor; PD, peritoneal dialysis; Hb, Hemoglobin; Alb, albumin; BUN, blood urea nitrogen; Scr, serum creatinine; SUA, serum uric acid; iPTH, intact parathyroid hormone; HbA1c, glycated hemoglobin A1c; LDL, low-density lipoprotein; TC, total cholesterol; TG, triglyceride; NT-proBNP, N-terminal pro-B-Type Natriuretic Peptide; Weekly PD Kt/V, weekly peritoneal dialysis fractional clearance index for urea; LVEF, left ventricular ejection fractions; IVS, interventricular septum; HR, hazard ratio; 95%CI, 95% confidence interval.

### The effect of ARNI on residual kidney function and peritoneal transport function

**Figure 4.**
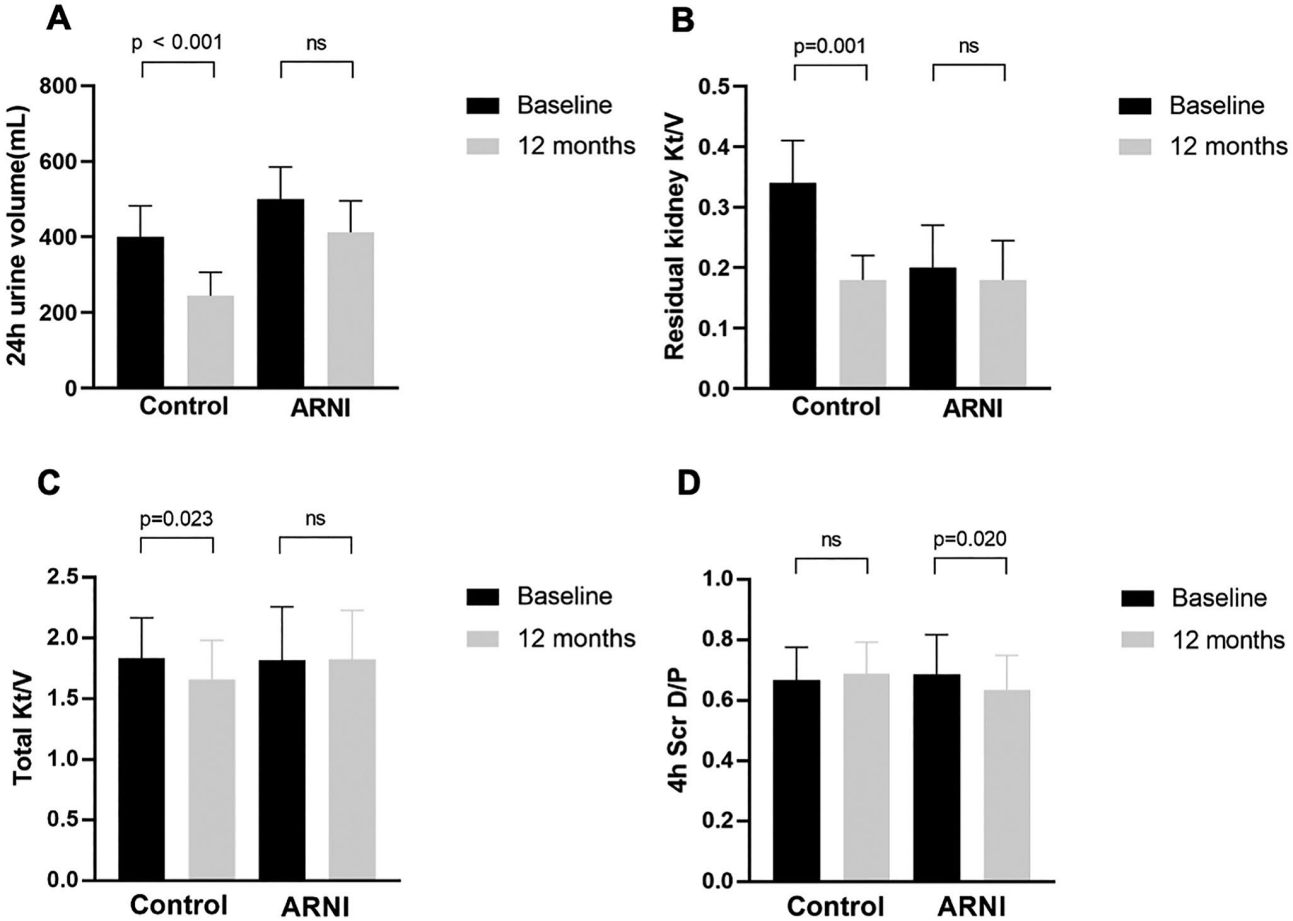
Changes in residual renal function and peritoneal function in the control group and the ARNI group at baseline and at 12 months. The histograms show the changes in residual renal function and peritoneal function at baseline and 12 months in both groups. (A) The change in 24h urine volume at baseline and 12 months in both groups. (B) The change in residual kidney Kt/V at baseline and 12 months in both groups. (C) The change in total Kt/V at baseline and 12 months in both groups. (D) The change in 4 h Scr D/P at baseline and 12 months in both groups. Mean ± SD presented for variables according with normal distribution while median with IQR presented for variables with abnormal distribution. Abbreviations: 24 h urine volume, 24 hours urine volume; Kt/V, urea clearance index; 4 h Scr D/P, 4-hour ratio of creatinine concentration in dialysate to plasma; *p* < 0.05 was considered to be statistically significant.

After 12 months of observation, the control group experienced a decrease in residual urine volume from 516 mL to 250 mL (*p* < 0.001), a decrease in residual kidney Kt/V from 0.368 to 0.156 (*p* = 0.001) and a decrease in total Kt/V from 1.835 to 1.661 (*p* = 0.023). In the ARNI group, there were no significant changes in residual urine volume, residual kidney Kt/V and total Kt/V from baseline. The residual renal Kt/V of PD patients in the ARNI subgroup showed no significant difference with or without adjustment of PD regimen (Table 2). The 4h Scr D/P ratio significantly decreased from 0.687 at baseline to 0.635 at 12 months (*p* = 0.020) (Figure 4).

### LCZ696 inhibits neoangiogenesis via the VEGFR2/ERK1/2 and Notch1 pathways

**Figure 5.**
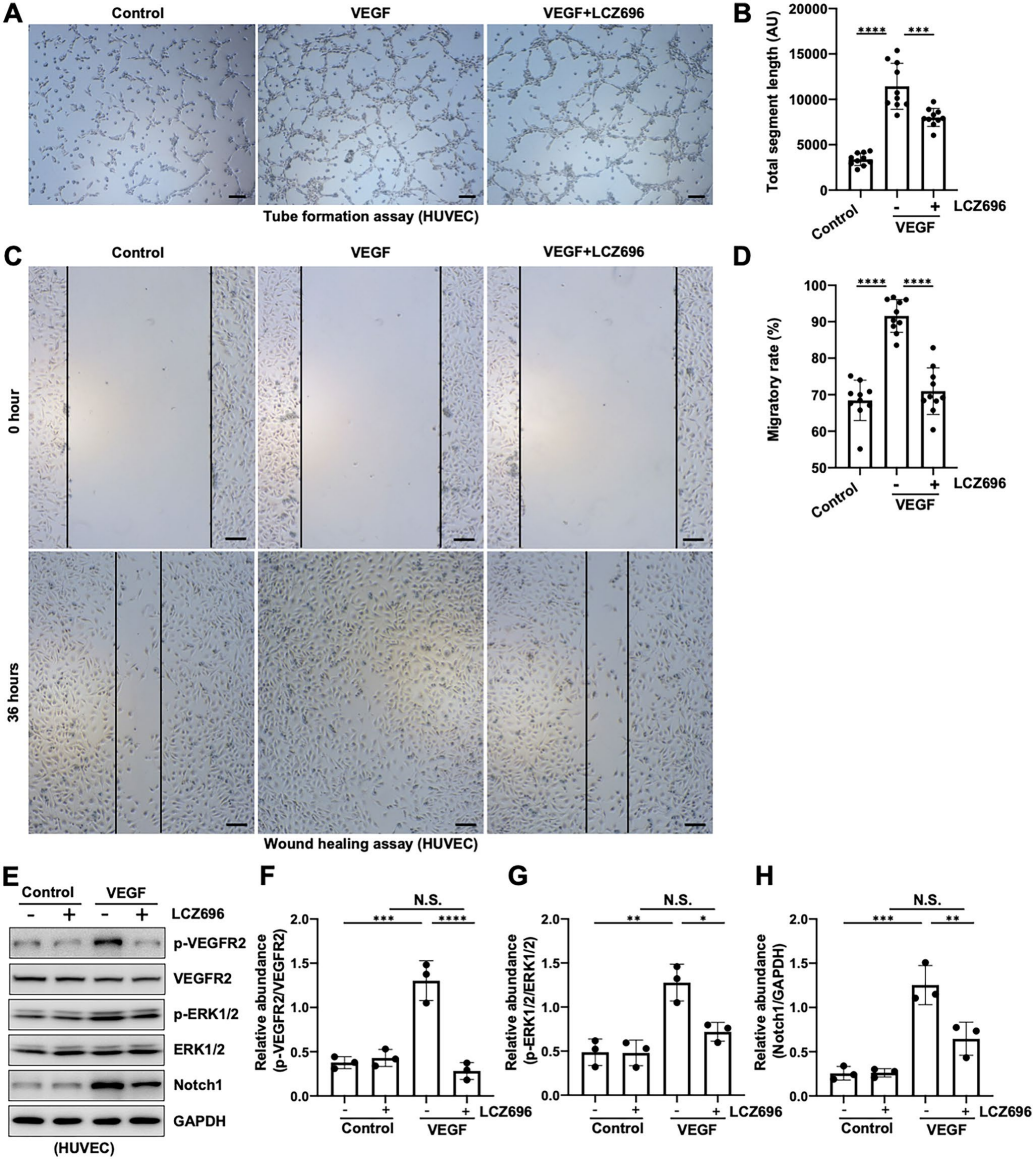
LCZ696 inhibits neoangiogenesis *via* the VEGFR2/ERK1/2 and Notch1 pathways. (A) Photomicrographs illustrating capillary networks of tubes formation in HUVECs. (B) Quantitative analysis of total segment length of tubes. (C) Wound healing assay of HUVECs at 0 h and 36 h. (D) Quantitative analysis of the width of the wound. (E) Western blot was conducted to evaluate the protein level of p-VEGFR2, VEGFR2, p-ERK1/2, ERK1/2, Notch and GAPDH in HUVECs cell lysates. (F) Scatter plot showing the densitometry analysis of p-VEGFR2 normalized by VEGFR2. (G) Scatter plot showing the densitometry analysis of p-ERK1/2 normalized by ERK1/2. (H) Scatter plot showing the densitometry analysis of Notch normalized by GAPDH. Data are expressed as mean ± SEM. *p < 0.05, **p < 0.01; *** *p* < 0.001; **** *p* < 0.0001. N.S., statistically not significant, with the comparisons labeled. All scale bars = 500 μm.

VEGF has strong neogenic effects and is a well-known stimulator for vasoformation [23], so we used VEGF (25 ng/mL) to induce angiogenesis in the current study. Compared with the control group, the cell tube formation and migration ability were dramatically increased in VEGF-induced HUVECs, while administration with LCZ696 significantly ameliorated this phenomenon (Figure 5(A–D)). Previous literature reported that VEGF combined with VEGFR2 can stimulate vascular endothelial cell proliferation and promote vascularization[24], thus we set out to examine whether LCZ696 inhibits neoangiogenesis *via* the VEGFR2 pathway. Immunoblot analysis showed that the expression of p-VEGFR2 and p-ERK1/2 were significantly increased in HUVECs stimulated by VEGF, administration with LCZ696 remarkably suppressed the activation of VEGFR2/ERK1/2 pathway. However, LCZ696 had no impact on the expression of total VEGFR2 and ERK1/2 (Figure 5(E–G)). Moreover, our results also showed that LCZ696 could inhibit the activation of the Notch1 signaling pathway, another important mechanism for promoting angiogenesis [18, 24] (Figure 5(E,H)). Overall, these data suggested that LCZ696 inhibits neoangiogenesis *via* the VEGFR2/ERK1/2 and Notch1 pathways.

## Discussion

In this multicenter retrospective study, we observed that ARNI is an independent factor that significantly reduces the occurrence of cardiovascular events in patients undergoing PD. Furthermore, it improved volume and echocardiographic parameters, protected residual renal function, and altered peritoneal transport characteristics. Subsequent *in vitro* experiments demonstrated that LCZ696, one of the active components in ARNI, can inhibit neoangiogenesis *via* the VEGFR2/ERK1/2 and Notch1 pathways. To date, there have been limited studies investigating the effects of ARNI on the cardiac function and prognosis of PD patients. Our study is the first to explore the prognosis of ARNI in PD patients by examining both clinical and molecular biological mechanisms.

The famous Determine Impact on Global Mortality and morbidity in Heart Failure (PARADIGM-HF) clinical study demonstrated that LCZ696 was associated with a 20% risk reduction for the primary composite endpoint (cardiovascular disease-related death and heart failure hospitalization) and a 16% reduction in all-cause mortality compared with enalapril among heart failure patients with normal renal function [5]. However, these large clinical prospective trials usually excluded end-stage kidney disease (ESRD) patients, although they are a common subset of patients in the real world. Some small-sample studies have observed the effect of ARNI on cardiovascular prognosis in dialysis patients. Chang et al. found that in a study including 10.9% of patients in CKD stage IV or V, ARNI use resulted in a significantly lower incidence of cardiovascular-related death or hospitalization during a 15-month follow-up period (33.4% vs. 56.6%; *p* = 0.001) [25]. The retrospective study by Liu et al. included 182 CHF patients on dialysis, and a total of 65 patients achieved the primary outcome of heart failure rehospitalization or all-cause mortality. The use of LCZ696 reduced heart failure rehospitalization rates (73.47% vs. 43.28%, *p* = 0.001), with no significant difference in mortality (8.96% vs. 10.2%) [26]. In our study, 19 patients achieved the primary outcome of adverse cardiovascular events during follow-up. In the subgroup study, we demonstrated that compared with RASi, the use of ARNI showed a trend toward reducing the incidence of cardiovascular events, with a statistically significant difference between the two groups (HR 2.811, 95%CI 1.063 − 7.434, *p* = 0.033), which was consistent with previous research findings. Multifactorial Cox regression analysis showed that the use of ARNI significantly reduced the occurrence of cardiovascular events, making it a protective factor for the occurrence of clinical outcomes. The results of all these studies confirm the effectiveness of ARNI in improving the cardiovascular prognosis of dialysis patients in the real world. A larger sample size, longer-term follow-up, and prospective cohort studies are needed to obtain more meaningful results.

PD patients often suffer from volume overload, which presents as edema, weight gain and severe hypertension [27]. Therefore, maintaining normal hydration is crucial in the management of patients with end-stage renal disease [28–30]. Neprilysin inhibition (NEPi) enhances the activity of the natriuretic peptide system producing natriuresis, diuresis, and inhibition of the renin-angiotensin system and sympathetic nervous system [31]. However, in our study, we did not observe a significant increase in ­peritoneal ultrafiltration volume among patients using ARNI. Instead, we found that these patients exhibited better-preserved residual renal function and achieved significant blood pressure control. This finding suggests that preserved renal function may play a crucial role in effectively improving volume status, which was consistent with previous studies [32–34]. The difference in observation time between studies may have contributed to this discrepancy [35]. It is important to note that it is well known that residual renal function gradually declines with the increase in dialysis duration. However, in our study, there was no statistically significant difference in baseline residual renal function between the two groups with different dialysis durations. This may be due to the small sample size or the presence of selection bias.

In our study, we found that ARNI has a significant advantage in improving LVEF and IVS. Heart failure is a clinical syndrome characterized by signs of volume overload such as edema and dyspnea, decreased cardiac contractility, and increased intracardiac pressure. The heart undergoes a series of structural changes after injury, which are clinically manifested as changes in the size, shape, and function of the heart [36]. A prospective single-group open-label study found that after 12 months of treatment with ARNI, LVEF increased from 28.2% to 37.8%, and left ventricular end diastolic volume index (LVEDVI) decreased from 86.93 to 74.15 mL/m^2^ in patients with heart failure with reduced ejection fraction (HFrEF). The left atrium volume index (LAVI) and E/e’ ratio also decreased significantly [37]. After 9 months of treatment with ARNI or ACEI, a cohort study of HFrEF patients found that LVEF increased from 24.9% to 36.4% with ARNI and from 28.7% to 40.5% with perindopril. Both ARNI and ACEI have a reversing effect on cardiac remodeling, but the effect of ARNI seems to be stronger due to the larger reduction in left ventricular end systolic volume (LVESV) (17.9 mL vs. 10.8 mL, respectively) [38]. One study included 49 patients (31 HD, 18 PD) who received at least 6 months of conventional renal replacement therapy with baseline LVEF ≤ 40%, 26 of whom were treated with LCZ696 and the remaining 23 with conventional therapy. The study showed a significant improvement in LVEF in the LCZ696 group compared with the control group (45.1% ± 11.7% vs. 31.3% ± 5.5%, *p* < 0.001), as well as an improvement in left ventricular diastolic function [39]. Our findings are consistent with previous studies, indicating that ARNI may ameliorate heart failure and improve cardiac function regardless of CKD stage. Given the complexity of the etiology of CKD and the limited research on the effect of ARNI on cardiac structure in dialysis patients, further validation with an expanded sample is still needed.

BNP was secreted by the ventricles in response to excessive stretching of myocardial cells and can play a crucial role in regulating blood pressure and blood volume. In comparison to BNP, NT-proBNP exhibited greater stability, lower biological activity, and was not influenced by neprilysin inhibitors. Therefore, the concentration of NT-proBNP decreased with effective treatment. NT-proBNP was considered as a useful marker in the diagnosis of patients with acute or chronic heart failure [20]. A meta-analysis included 27 studies indicated that elevated NT-proBNP levels predicted higher risk of all-cause cardiovascular mortality and cardiovascular events in ESRD patients [21]. In our analysis, we observed that elevated NT-proBNP was an independent risk factor for cardiovascular prognosis. Although some studies have suggested that the only pathway for NT-proBNP clearance is glomerular filtration, recent studies have demonstrated the limited effect of eGFR on NT-proBNP in patients with renal insufficiency. These studies indicate that diagnostic thresholds can be adjusted based on the level of renal function [40–42]. Studies have confirmed that NT-proBNP levels can still effectively predict the risk of cardiovascular events and mortality when ESRD-specific thresholds of elevation are used [21].

Multiple studies indicate that a high peritoneal solute transport rate (PSTR) leads to volume overload, poor nutritional and metabolic status, and increased mortality. It is now generally thought that a fast PSTR depends on the effective surface area of capillaries and their permeability and peritoneal blood circulation [43]. Patients with fast PSTR tend to chronically absorb glucose from the dialysate, leading to obesity, hyperglycemia, hyperinsulinemia, and hyperlipidemia, which may contribute to the development of insulin resistance and metabolic syndrome [44]. Additionally, high glucose peritoneal dialysis fluid (PDF) induces the formation of glucose and glucose degradation products (GDPs) and advanced glycation end products (AGEs), resulting in a significant increase in vascular density, altered peritoneal transport state, and peritoneal inflammation [45]. Vascular endothelial growth factors (VEGFs) play a crucial role in angiogenesis. Previous studies have indicated a significant relationship between plasma and dialysate VEGF levels and PSTR, suggesting that inflammation, angiogenesis, and ­peritoneal transport may be interrelated and involved in the pathophysiology of high PSTR in PD patients [46]. Furthermore, the epithelial-to-mesenchymal transition (EMT) can also influence vascularization. When mesothelial cells (MCs) undergo EMT, non-epithelioid MCs produce higher amounts of VEGF and are linked to a high peritoneal transport rate [47]. In our study, we demonstrated that ARNI can decrease 4h Scr D/P. We also verified through *in vitro* experiments that LCZ696 effectively inhibits angiogenesis *via* the VEGFR2/ERK1/2 and Notch1 pathways. This may be one of the mechanisms by which ARNI improves peritoneal transport function in PD patients. Therefore, ARNI’s application in PD patients might protect the peritoneum by inhibiting angiogenesis. This is the first study to confirm ARNI’s effect on the peritoneum in PD patients, showing that ARNI can inhibit angiogenesis, thereby reducing solute transport. Intervening in these pathways can effectively inhibit abnormal angiogenesis, providing new treatment strategies for diseases like peritoneal fibrosis. Previous study confirmed that high-transporter patients are at an increased risk of atherosclerosis when compared with their low-transporter counterparts through chronic inflammation [48]. Another study showed that transport rates did not have a significant effect on biochemical parameters or cardiac structural/functional parameters in pediatric PD patients [49]. Hence, the impact of peritoneal transport characteristics on cardiovascular outcomes remains an area that warrants further exploration.

Our study has identified several limitations. Firstly, this was a multi-center retrospective study with a relatively small sample size, which limited the standardization of patients’ clinical data using a uniform testing tool. If certain biochemical indices fluctuate over time and are not analyzed as time-dependent variables, such as we were not able to accurately record PD regimen adjustment, this may potentially impact the results. Secondly, retrospective studies inherently possess certain unavoidable confounding factors, such as the etiology of chronic kidney disease, the glucose exposure and dialysis vintage, as well as the lack of control over factors that may influence the outcome during the observation period. Thirdly, we excluded the patients who had been using ARNI for less than 1 year and those who using automated PD, which may carry a risk for selection bias. However, it is noteworthy that our study is the pioneering investigation into the effects of ARNI on cardiac and peritoneal function in PD patients through both human and *in vitro* experiments.

## Conclusions

ARNI may potentially have a role in protecting the heart and can decrease solute transport in PD patients. Larger, randomized, double-blind studies are needed for more conclusive evidence.

## Supplementary Material

figure_4.tiff

figure_2.tiff

figure_1.tiff

figure_3.tiff

figure_5.tiff
