# Supplementary figures and images for: The impact of angiotensin-receptor neprilysin inhibitors on cardiovascular events and solute transport function in peritoneal dialysis patients: a multicenter retrospective controlled study
Source: Ren Fail. 2024 Nov 28;46(2):2431637. doi: 10.1080/0886022X.2024.2431637 (PMC11610316; doi:10.1080/0886022X.2024.2431637)

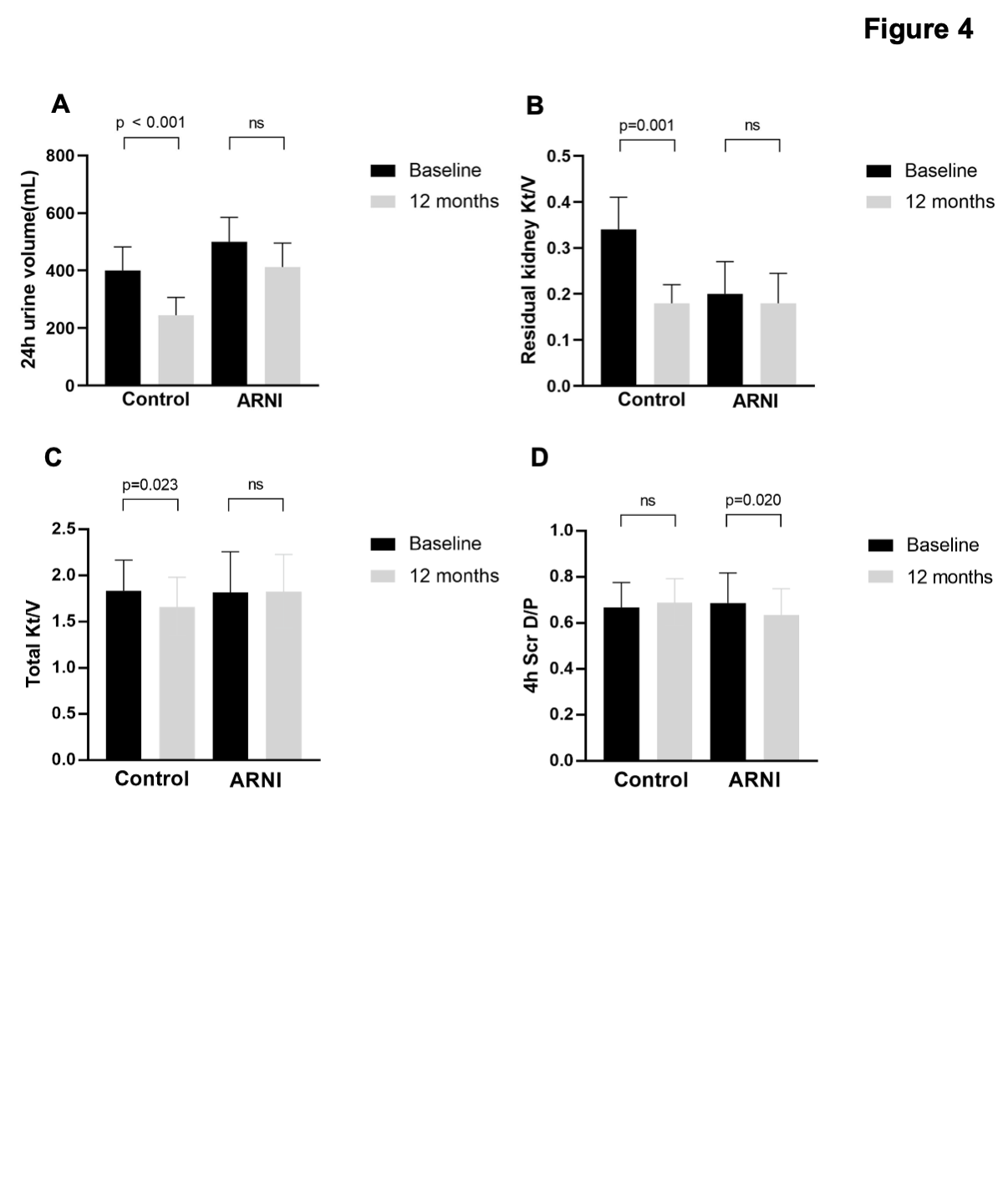

Supplement: figure_4.tiff [file IRNF_A_2431637_SM8508.tiff]

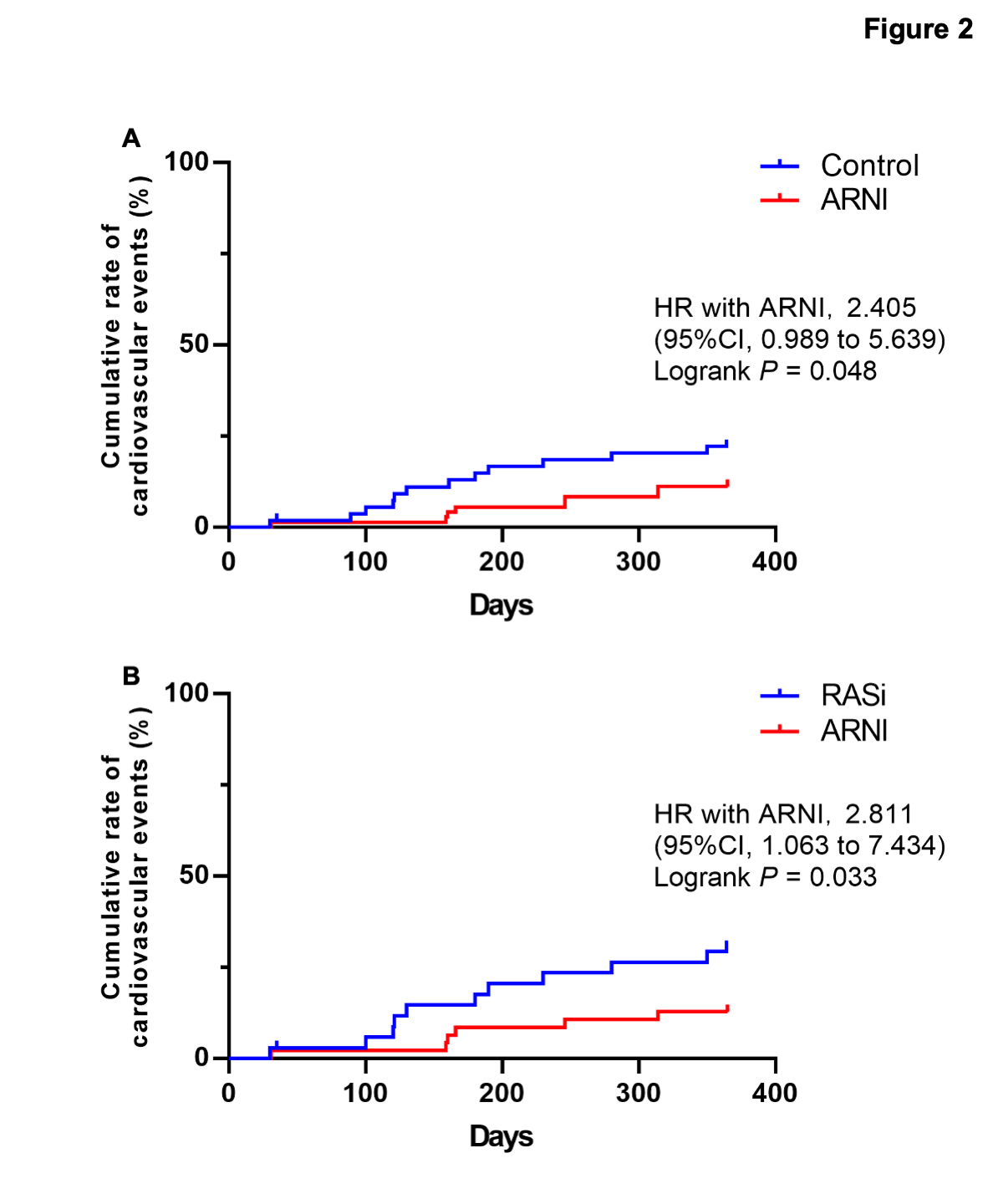

Supplement: figure_2.tiff [file IRNF_A_2431637_SM8507.tiff]

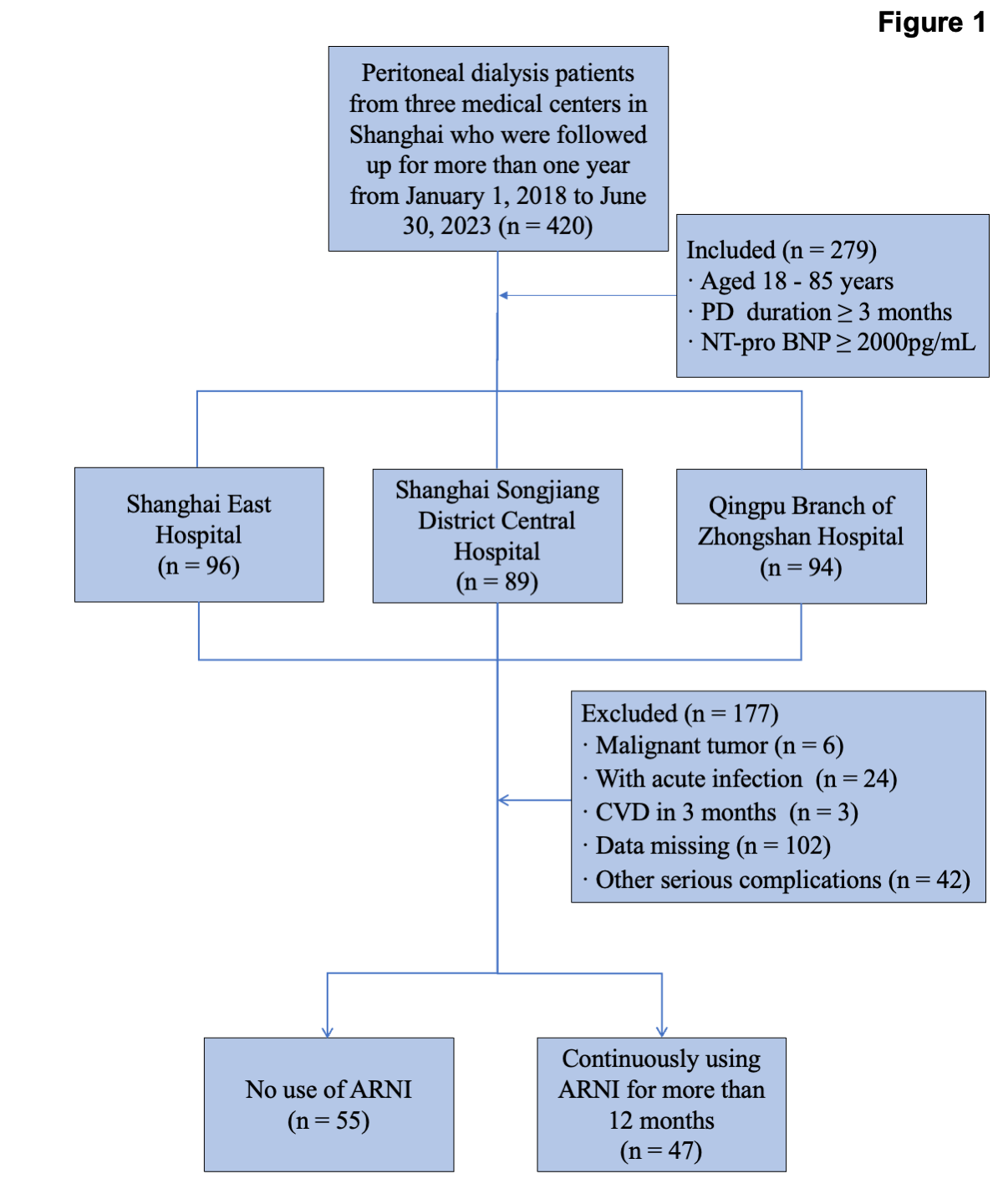

Supplement: figure_1.tiff [file IRNF_A_2431637_SM8506.tiff]

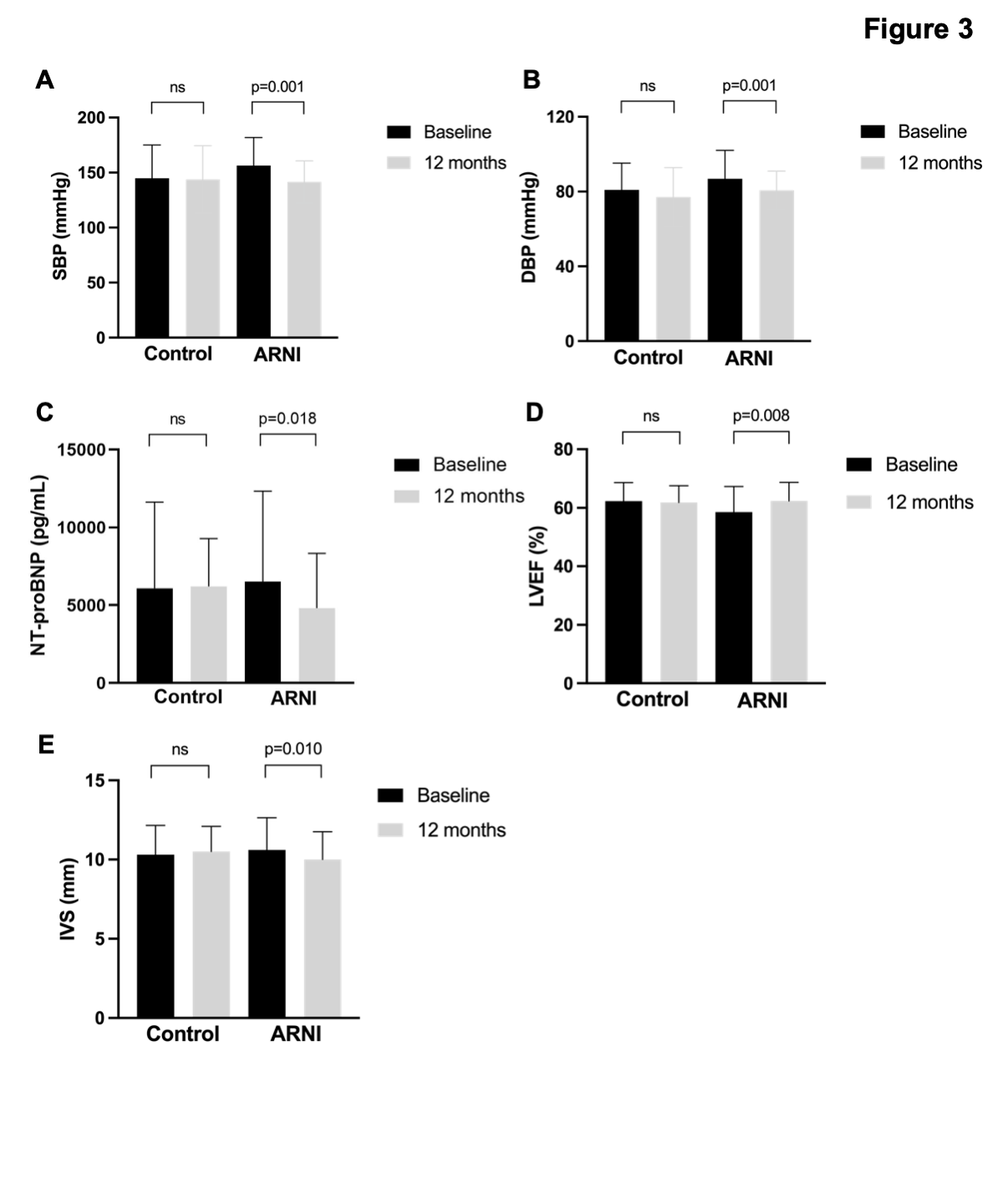

Supplement: figure_3.tiff [file IRNF_A_2431637_SM8505.tiff]

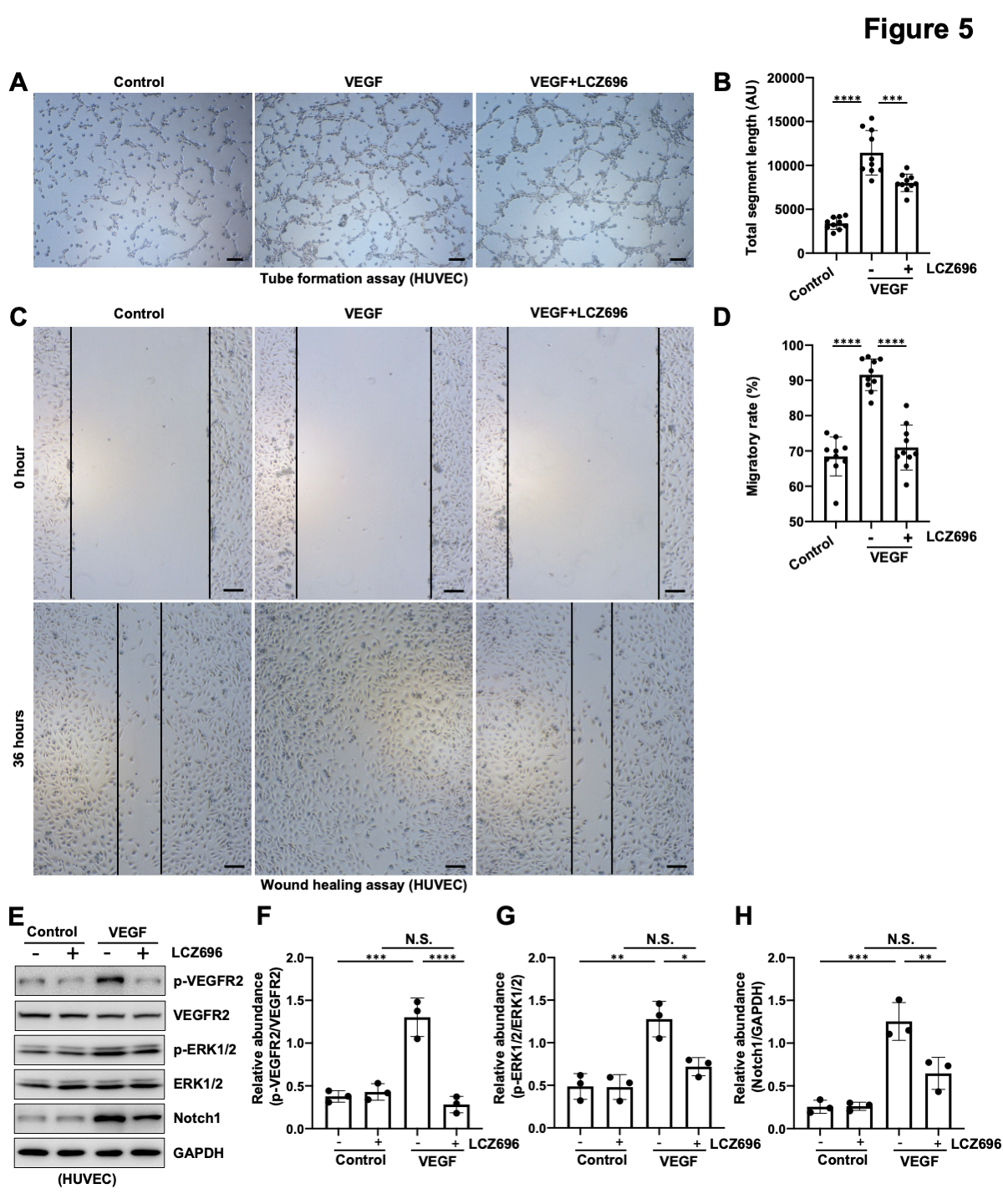

Supplement: figure_5.tiff [file IRNF_A_2431637_SM8504.tiff]
